# Correction: Towards Developing an Initial Programme Theory: Programme Designers and Managers Assumptions on the Antiretroviral Treatment Adherence Club Programme in Primary Health Care Facilities in the Metropolitan Area of Western Cape Province, South Africa

**DOI:** 10.1371/journal.pone.0166507

**Published:** 2016-11-09

**Authors:** The PLOS ONE Staff

## Notice of Republication

This article was republished on October 21, 2016 to correct errors in figures that were introduced during the typesetting process. The publisher apologizes for the errors. Please download this article again to view the correct version. The originally published, uncorrected article and the republished, corrected article are provided here for reference.

## Supporting Information

S1 File Originally published, uncorrected article. (PDF)

S2 File Republished, corrected article. (PDF)
