# Supplementary material for: Correction: Towards Developing an Initial Programme Theory: Programme Designers and Managers Assumptions on the Antiretroviral Treatment Adherence Club Programme in Primary Health Care Facilities in the Metropolitan Area of Western Cape Province, South Africa
Source: PLoS One. 2016 Nov 9;11(11):e0166507. doi: 10.1371/journal.pone.0166507 (PMC5102467; doi:10.1371/journal.pone.0166507)
Supplement: S1 File — (PDF) [file pone.0166507.s001.PDF]

PLOS | ONE

RESEARCH ARTICLE

Towards Developing an Initial Programme Theory: Programme Designers and Managers Assumptions on the Antiretroviral Treatment Adherence Club Programme in Primary Health Care Facilities in the Metropolitan Area of Western Cape Province, South Africa

Ferdinand C. Mukumbang[1,2]*, **Sara van Belle**[2,3], **Bruno Marchal**[1,2], **Brian van Wyk**[1]

1 School of Public Health, University of the Western Cape, Bellville, Western Cape Province, South Africa, **2** Department of Public Health, Institute of Tropical Medicine, Antwerp, Belgium, **3** Institute of Development and Management, University of Antwerp, Antwerp, Belgium

* mukumbang@gmail.com

CrossMark
click for updates

OPEN ACCESS

Citation: Mukumbang FC, van Belle S, Marchal B, van Wyk B (2016) Towards Developing an Initial Programme Theory: Programme Designers and Managers Assumptions on the Antiretroviral Treatment Adherence Club Programme in Primary Health Care Facilities in the Metropolitan Area of Western Cape Province, South Africa. PLoS ONE 11 (8): e0161790. doi:10.1371/journal.pone.0161790

Editor: Omar Sued, ARGENTINA

Received: May 5, 2016

Accepted: August 11, 2016

Published: August 25, 2016

Copyright: © 2016 Mukumbang et al. This is an open access article distributed under the terms of the Creative Commons Attribution License, which permits unrestricted use, distribution, and reproduction in any medium, provided the original author and source are credited.

Data Availability Statement: The data set supporting the results of this article is available in the figshare repository, doi: 10.6084/m9.figshare.3122659 and hyperlink to dataset in https://figshare.com/s/da07e7a1eb44d562b7e6.

Funding: The PhD from which this study emanated was funded by the Medical Research Council of South Africa in terms of the National Health Scholars Programme from funds provided for this purpose by the National Department of Health, http://www.mrc.ac.za/. This research was partially funded by an African

Abstract

Background

The antiretroviral adherence club intervention was rolled out in primary health care facilities in the Western Cape province of South Africa to relieve clinic congestion, and improve retention in care, and treatment adherence in the face of growing patient loads. We adopted the realist evaluation approach to evaluate what aspects of antiretroviral club intervention works, for what sections of the patient population, and under which community and health systems contexts, to inform guidelines for scaling up of the intervention. In this article, we report on a step towards the development of a programme theory—the assumptions of programme designers and health service managers with regard to how and why the adherence club intervention is expected to achieve its goals and perceptions on how it has done so (or not).

Methods

We adopted an exploratory qualitative research design. We conducted a document review of 12 documents on the design and implementation of the adherence club intervention, and key informant interviews with 12 purposively selected programme designers and managers. Thematic content analysis was used to identify themes attributed to the programme actors, context, mechanisms, and outcomes. Using the context-mechanism-outcome configurational tool, we provided an explanatory focus of how the adherence club intervention is roll-out and works guided by the realist perspective.

Results

We classified the assumptions of the adherence club designers and managers into the roll-out, implementation, and utilisation of the adherence club programme, constructed around

Doctoral Dissertation Research Fellowship (ADDRF) award offered by the Africa Population and Health Research Center (APHRC) in partnership with the International Development Research Centre (IDRC), http://aphrc.org/.

Competing Interests: The authors have declared that no competing interests exist.

the providers, management/operational staff, and patients, respectively. Two rival theories were identified at the patient-perspective level. We used these perspectives to develop an initial programme theory of the adherence club intervention, which will be tested in a later phase.

Conclusion

The perspectives of the programme designers and managers provided an important step towards developing an initial programme theory, which will guide our realist evaluation of the adherence club programme in South Africa.

Background

Starting from a few isolated HIV cases in the late 1980s, South Africa had one of the fastest infection rates in the world, reaching the 7 million mark at the end of 2015 [1]. The South African Ministry of Health responded to the HIV epidemic by rolling out antiretroviral drugs at no direct financial cost to patients in 2004 and subsequently decentralising antiretroviral care and treatment to primary health care facilities nationwide [2]. Through this strategy, an estimated 3.1 million (32.2%) people living with HIV/AIDS (PLWHA) in South Africa have been initiated on antiretroviral treatment (ART) as of April 2015 [3].

The success in enrolling patients on antiretroviral therapy (ART) has led to increasing numbers of patients in long-term care, which in turn contributes to clinic saturation [4]. As facilities became more congested, patient waiting times increased, patients received less individual attention, and the risk of staff burnout increased [5–9]. These circumstances have been associated with poor retention of patients and sub-optimal adherence to ART [10–12].

To address the issues of poor retention in care and sub-optimal adherence to medication, various community-based (group- and individual-based) approaches to improve HIV care and treatment have been implemented across sub-Saharan Africa. Group-based models of ART care and treatment work on the principle of grouping stable patients for decentralised care at lower levels (task-shifting), providing peer support among patients, reducing appointment frequency, separating the drug-delivery from clinical visits, making care simple and user-friendly, and creating a conducive environment for health education for group members [13–15]. One such group-based model for the treatment and care of ART patients is the "antiretroviral treatment adherence club, otherwise known as "adherence club" or "ART club", which was selected as the best practice model for retaining ART patients in care [16]. The antiretroviral adherence club intervention has been described as the adherence club or the ART club. When the intervention was originally conceived, it was called the 'Adherence Clubs' model of care, which was renamed 'ARV Chronic Clubs' model during the first phased rollout. To suit the requirements of the Western Cape Provincial Department of Health, the name of the intervention was changed from MSF's original '*Adherence Clubs*' to `*ARV Chronic Clubs*'. For consistency will refer to it here as adherence club.

The adherence club, a health system intervention, was conceived, designed, and implemented through a partnership between the Western Cape Provincial Department of Health (WC DoH), the Treatment Action Campaign (TAC), the Cape Town Municipality City Health department (CoCT DoH), Médecins Sans Frontières (MSF), and the Institute for Healthcare Improvement (IHI). MSF offered expertise, IHI provided quality improvement (QI) expertise and the WC DoH offered the political clout. A steering committee of senior managers was

formed and all the groups identified above collaborated closely towards the project design and execution [4].

The adherence club intervention was designed to improve retention in care and adherence among 'stable patients' on ART in primary health care facilities in the Western Cape province [16]. Stable patients are defined as patients aged 18 years or more, on the same ART regimen for at least 12 months, with the two most recent consecutive viral loads of the patient undetectable, and having no medical condition requiring regular clinical consultations more than once a year. Between 2007 and 2011, pilot programmes of the adherence club model in selected primary health care facilities in the Western Cape Province demonstrated improved patient flow within the clinic, increased monthly enrolment of new patients, better adherence to medication, and decreased loss to follow-up rates [4,17,18].

In 2011, the adherence club intervention was officially rolled out for the first phase of implementation in selected public health care facilities within the metropolitan area of the Western Cape Province. During this phase, a sub-group comprising of 14 of the 25 large ART facilities were selected based on their size, willingness to participate, and the availability of a programme manager to support the facility during the project. By the end of 2014, adherence clubs were rolled out to an estimated 300 facilities in the province [18]. Although the routine HIV/AIDS registry indicated sustained retention in care and optimal adherence rates (viral suppression) amongst ART patients in adherence clubs, anecdotal reporting suggested implementation challenges in the start-up and day-to-day running of the adherence clubs in some facilities. This reflected in the variation of retention in care and adherence rates obtained from routine monitoring and evaluation data.

In consultation with the Western Cape Provincial Department of Health's HIV/AIDS, Sexually Transmitted Infections, and Tuberculosis (HAST) programme, an evaluation of the adherence club programme was undertaken. The realist evaluation approach was chosen because it holds potential to answer questions about what works, for whom, and under what contextual circumstances in a complex programme such as the adherence clubs [19].

Methodology

Theoretical framework

Realists seek to understand how and why a programme works, for whom, and in what circumstances [20]. The philosophical basis of realist evaluation is realism, which assumes that an external reality can be assessed through configurations of contexts, mechanisms and outcomes [21]. Therefore, realist evaluation, through its articulation of a configured context-mechanism-outcome (CMO) in its findings, provides an explanatory focus which seeks to understand and interrogate "how, why and for whom a programme works?" in programme evaluation. It is generally acknowledged, therefore, that evaluation approaches such as realist evaluation can potentially open the "black box" of programme mechanism and provide greater insights on programme causality [22]. Typically, realist evaluations start with an initial programme theory (hypothesis) and end with a more refined theory.

A programme theory encompasses the assumptions and perspectives of the programme designers and implementers and is assumed to underlie a particular intervention [20]. Programme theories can be defined as the set of assumptions of programme designers (or other actors involved) that explain how they expect the intervention to achieve its objective(s) [23]. Describing the often implicit set of assumptions that steer the choice and design of a programme or intervention is useful because it allows investigators to understand what is being implemented and why. To elicit the programme theory, researchers unearth the models that the actors are implicitly using to describe and understand the intervention–what Pawson and

Tilley [20] call 'folk theories'–through individual interviews or group discussions [23]. Additional information may be derived from analysis of programme documents and/or policy documents. Finally, evidence and knowledge from other similar programmes may be used to build a complete programme theory. Testing the programme theory through empirical research entails identification of Context-Mechanism-Outcome (CMO) configurations, an analytic instrument, to help build the programme theory [24]. This leads to specification of the programme theory. At the end of a realist evaluation cycle, the evaluator obtains a refined programme theory, while at the end of multiple realist evaluation cycles of a particular type of intervention, the evaluator gradually builds up towards a middle range theory.

In summary, exploring what programme designers and managers think about how the adherence club intervention works means seeking an understanding of how these stakeholders understand the influence of various contextual elements, what mechanisms are in action, and how the adherence club would trigger these mechanisms in specific contextual conditions that contribute to the outcomes. The results of this exercise are then combined with the results of a systematic review to formulate the initial programme theory that will be tested in subsequent empirical studies of adherence clubs in the Western Cape Province.

The first step in conducting a realist evaluation is to elicit the programme theory that explains how the intervention is expected to work according to the programme designers and implementers [20]. An important step in eliciting the programme theory involves eliciting the assumptions and perspectives of the programme designers and managers with regard to how and why the adherence club intervention would work. In this article, we report on the assumptions of the adherence club programme designers and managers (implementers) regarding their understanding of the programme implementation process, how and why it would achieve the anticipated outcomes, and under what conditions. We also discuss how this informed our development of the initial programme theory of the adherence club intervention.

Methods

We used an exploratory qualitative study design. Two methods of data collection were adopted: a review of policy documents and other literature on the adherence club programme; and semi-structured in-depth interviews with programme designers and managers (implementers).

A review of policy documents and other literature on adherence club programme. We obtained a variety of documents regarding the intervention (e.g. implementation reports, data files, and other written artefacts) from different sources with the purpose of collecting independently verifiable data and information about the ART adherence clubs [25]. The document review served two purposes. First, reviewing existing programme documents helped us to understand the history, evolution, and operation of the adherence club programme. Secondly, the information obtained from the documents served as pointers to the aspects that required more probing during the interview process with the key informants.

The first author searched various databases (PubMed, Google search, Google Scholar, and EBSCOhost) and relevant websites (Médecins Sans Frontières, the provincial Department of the Western Cape and Health E-news) for documents on the ART adherence club intervention. He used search terms such as "adherence club", "ART adherence club", "ART clubs", "facility-based adherence club", and "MSF innovation in ART management in South Africa". This search process identified documents such as programme descriptions, implementation guidelines, and a toolkit on the adherence club. Policy documents for the rollout and implementation of the adherence clubs were obtained from the HAST Directorate of the Western Cape Province. After the search process, we judged each document for relevance (are the claims made based on the relevant and appropriate information?) and utility (are the knowledge

claims appropriate to our needs?) [26,27]. Twelve (12) documents were included in the document review. Table 1 below shows the types and description of the documents included in the document review.

The documents were analysed using the document analysis process described by Bowen [25]. We developed a data code manual a priori (Table 2), based on the realists' understanding of actors, context, mechanism and outcome. The data code manual was designed to guide the initial data analysis and extraction of information on the relevant actors, the context, the shifts in dispositions–thought process and decisions–of participants (mechanisms) during the implementation of the intervention, and the identified outcomes related to the intervention (immediate, intermediate and long-term).

Semi-structured in-depth interviews with designers and implementers. After the document review process, FCM and BVW conducted face-to-face semi-structured in-depth interviews with purposively selected key informants–persons who were involved in designing the adherence club intervention, and people working on the implementation or management of the adherence club programme and its rollout. According to Leeuw [28], managers, stakeholders, and workers involved in a programme have "cognitions" (or "mental maps") about the organisation and the environment of the programme. Realist evaluators can articulate the opinions and perspectives of the programme designers and managers through (a) general conversations and (b) interviews with a more limited and purposive selection of stakeholders [29]. Our goal was to further develop the theories from the document review, with both methods (document review and interviews) serving a 'conceptual refinement function' (theory gleaning) [21]. For this purpose, we explored the different perspectives of the key informants and identify the key similarities and differences in how they viewed the intervention and how they considered the intervention to achieve its objectives.

A total of 12 participants from different organisations working on various aspects of the adherence club at different levels of the implementation chain were purposively recruited in the study. We identified the positions of individuals whom we thought would be key informants. These participants were asked to recommend others whom they thought would be relevant key informants (snowballing). While conducting conventional qualitative studies, recruitment of study participants is continued until no new relevant knowledge is obtained (thematic saturation) [30]. The idea of standardizing saturation as a means of obtaining the appropriate sample size is challenged by O'Reilly and Parker [31]. Therefore, in this realist evaluation process, confirmation of the realist hypothesis was based on relevance and rigour rather than thematic saturation [24]. It should be noted that the health facility managers and operational staff involved in the actual execution of the programme were not included in this phase of the study.

An interview guide was developed for conducting the interviews (S1 File). The interview guide included questions related to the design of the adherence clubs, the original idea behind the design, the goals and objectives, and the various components that make up the adherence club intervention. The interview questions also sought to obtain insights into the context required for the adherence club to have the expected impact, the dynamics that the adherence club brings to the treatment and care of people living with HIV/AIDS, and the possible mechanisms of change. Although English is not the native language of all the participants, they could understand and express themselves fluently for the interviews to be conducted.

We applied the 'realist interview technique' whereby theories are placed before the interviewees for them to comment with the view to provide refinement [20]. This method suggests that the interviewer teaches the interviewee the particular programme theory under consideration and then the respondent being the 'expert', in turn, is able to teach the interviewer about the components of the programme in an informed way (learner-teacher cycle) [32]. By this

Table 1. A description of the documents that were included in the document review.

Title	Author/year	Document Type	Description
The adherence club toolkit	MSF/WCPG (2013)	Toolkit	This toolkit provides a detailed account on how to establish clubs, the ART club staff organogram, lessons learned through the Khayelitsha implementation experience and tools utilised in the ART club model.
Adherence club register	MSF/WCPG	Adherence Club register	This document is the adherence club register that the club facilitator fills in during every club meeting.
ART adherence clubs: A long-term retention strategy for clinically stable patients receiving antiretroviral therapy	Wilkinson LS (2013)	Journal Article	This article describes the adherence club programme (structure and function). It also elaborates on the implementation strategy that was employed and provides the experiences from the implementation of the adherence clubs in Khayelitsha.
Treating Millions for HIV—The Adherence Clubs of Khayelitsha	Champion EW (2015)	Journal Article	This article describes the experiences of the author as he investigated the functioning of a community-based adherence club in the home of a club member. He also reports on an interview that he had with the coordinator of the adherence club programme for MSF.
Out-of-clinic adherence club for delivery of ARVs shows better retention than standard of care	Odendal L (2012)	News Article	This article discusses the advantages of the adherence club programme over the standard clinic care with regard to retention in care and adherence. It elaborates on a comparative study that was conducted to investigate the effectiveness of the adherence club, the findings of the study and the implications.
Reaching closer to home: Progress implementing community-based and other adherence strategies supporting people on HIV treatment	SAMU & MSF (2013)	Report	This document describes the progress that has been made in implementing community-based models of ART care since the release of the report "Closer to Home" by UNAIDS and MSF in July 2012.
MSF again paves the way with ART	Bateman C (2013)	Journal Article	This article provides a general description of the adherence club, emphasising the superiority of the adherence club model of care over the standard clinic care. The article states some conditions that are necessary for the adherence club initiative to be successful. The article ends by providing a doctor's perspective on the adherence clubs.
Clubbing together for treatment	Health-e News (2012)	Health News Article	This article describes the adherence club intervention and its role in reducing patient loads (ART initiation). It discusses the effectiveness of the adherence club, and how this could be replicated in other areas.
Western Cape ART-Adherence Treatment Clubs and Preventative Therapy for New-borns	WCG (2014)	News Article	This news article was written on the inauguration of the World AIDS day on December 2014. It describes the progress that has been made on the retention in care of PLWHA since the inception of the adherence clubs. It also reports on the progress made in the implementation of the adherence club in the Western Cape Province.
Guidelines for ART clubs	Western Cape Government (2015)	Standard Operating Practice (SOP) of the adherence club	This document describes the standard operating practices of the adherence club. It starts by describing the aims and the objectives of the adherence club, outlines the requirements to establish the adherence club, the organisation and running, the pharmacy requirements for the scripting and dispensing medication to ART patients and finally, the scripting schedule of the ART medication.
Implementation scale up of the Adherence Club model of care to >30,000 stable ART patients in the Cape Metro, South Africa 2011–2015	Wilkinson, L. et al. (2015)	Conference presentation and Journal article	This presentation focuses on the nature of the adherence club and its impact on the retention in care rates of PLWHA. It also describes the possible different types of adaptation of the programme.

(*Continued*)

Table 1. (*Continued*)

Title	Author/year	Document Type	Description
Closer to home: Delivering antiretroviral treatment therapy in the community: Experiences from four countries in Southern Africa	MSF/UNAIDS (2012)	Report	This paper describes the implementation and the results of community-based methods of delivering ART in communities in four Southern African countries.

doi:10.1371/journal.pone.0161790.t001

method, we presented tentative CMO configurations of the programme (developed from the document review process) and encouraged the respondents to explain their views about the tentative CMOs [33]. We also used trigger questions to obtain specific information. Feedback was later provided to the interviewees on the concepts and theories developed from their comments and contributions for validation. This process is described by Leeuw as the "elicitation cycle" [28]. The interview process was conducted between October 2015 and February 2016. Table 3 below shows the various stakeholders who were included in the key informant interviews.

Data Management

For identification of the information source and anonymity, the key informant interviews are coded as KII_x where x represents an arbitrary number from 1–12. In a similar manner, information obtained from a document are coded as 'DR_x' for document review and 'X' for the document number.

Data Analysis

We applied a realist philosophical 'lens' to the data analysis process, applying the CMO configurations as the analytical tool. The goal of the analysis process at this stage was to identify CMO configurations that represent the vision, goals and thought processes of the adherence club programme designers and managers (implementers). Information from both the document analysis and the semi-structured in-depth interviews were used to formulate CMO configurations that would inform the further development of the adherence club programme

Table 2. Data code manual.

Category		Definition	Coding Rules
**Actors**		These are the individuals, groups, and institutions who play a role in the implementation and outcomes of an intervention	This was coded as the actions or actual practices of an individual, group or institution.
**Context**		Context refers to salient conditions that are likely to enable or constrain the activation of programme mechanisms.	Components of both the physical and the social environment that favour or disfavour the expected outcomes
**Mechanisms**		This refers to any underlying determinants or social behaviours generated in certain contexts	Any explanation or justification why a service or a resource was used by an actor to achieve an expected outcome, or considered as a constraint
**Outcomes**	Immediate outcome	Describes the immediate effect of the adherence club programme activities	Immediate outcome typically refers to changes in knowledge, skills or awareness, as these types of changes typically precede changes in behaviours or practices.
	Intermediate outcome	Intermediate outcomes refer to behavioural changes that follow the immediate knowledge and awareness changes.	Codes here define a move from direct outcomes to intermediate outcomes, identified through the indirect impact of the activity and accountability of the programme.
	Long-term outcome	Refer to change in the medium- and long-term, such as a patient's health status, and impact on community and health system	The codes here represent the further indirect impact of the activity demonstrating the lesser accountability of the programme.

doi:10.1371/journal.pone.0161790.t002

Table 3. List of key informants interviewed.

Stakeholder	Number of participants	Number of interviews per group of participants
Médecins Sans Frontières	2	3
Treatment Action Campaign	1	1
Western Cape Provincial HAST directorate	1	2
Sub-structure HAST Managers	3	4
Sub-structure HAST MOs (Medical Officers)	3	4
The City of Cape Town	1	2
Institute for Health Care Improvement	1	1
Total	12	17

doi:10.1371/journal.pone.0161790.t003

theory. This process was first applied to the document analysis for two purposes: (a) to identify gaps and (b) for the identification of themes and the construction of the tentative CMO configurations

During the final data analysis, information from both sources was pooled to formulate the CMOs. The analysis process was conducted in two phases: Phase 1 involved identifying the context, mechanisms and outcomes, as well as the actual interventions and the actors involved from the document review process and the in-depth interviews (thematic content analysis). Phase 2 entailed synthesising the empirical findings, which involved exploring how the context, mechanisms and outcomes are linked (generative explanation).

Phase 1: Theme identification

We employed the thematic content analysis method [34] for identifying, analysing, and reporting the CMO themes within data [35]. This approach fits in the realist analysis logic [36].

First, we transcribed the interviews verbatim. Both transcripts and documents were fed into the Atlas.ti version 7 software for a systematic management of the data. Secondly, we read the transcripts and documents repeatedly to familiarise ourselves with the contents. We applied the coding framework (**Table 2**) and coded the text. We specifically searched for instances where respondents talked about influential context, underlying actors' behaviour, possible mechanisms, and expected outcomes (initial, intermediate, and long-term). We sorted the segments of the texts and applied a process of data retrieval to organise the codes according to the different segments (context, mechanism, and outcomes).

Phase 2: Data synthesis—Context-Mechanism-Outcome (CMO) configurations

A realist approach of how an intervention is expected to work as anticipated by the programme designers and managers (expressed in 'folk theories') should explain how they believe the various components of the programme, the context, the actors and the mechanisms are linked and interacting to produce the expected outcome(s). Linsley, Howard, and Owen suggest that the explanation should consist of propositions about how the interplay between structure and agency in a social action constituted the regularity [37].

The complexity of the programmes and the situations in which they are embedded often make it difficult to anticipate the ways in which change might be brought about by the multiple stakeholders and their myriad (potential) responses [38]. After we identified the themes related to actors, context, mechanism and outcomes of the adherence club intervention from the texts,

following the recommendation of Wong et al. [39], we formulated preliminary explanations (causal inferences) of how the adherence club intervention could be successfully implemented, and how it could achieve the expected outcomes using the context, mechanism, and outcome components.

We grouped the explanatory ingredients (context, mechanism, and outcomes) in the form of CMO configurations [20], the analytic centrepiece of realist evaluation [40]. After formulating the preliminary CMO configurations, we applied an iterative consultative process with five of the previously interviewed key informants to understand how the actors, mechanism, and context are expected to link to each other to produce the outcome(s). The emphasis at this stage was to obtain plausible causal explanations in the form of CMOs [41].

Ethical Considerations

This study is part of a larger project "A realist evaluation of the antiretroviral treatment adherence club programme in selected primary health care facilities in the metropolitan area of Western Cape Province, South Africa" which has received ethics clearance from the University of the Western Cape Research Ethics Committee (UWC REC) (Registration No: 15/6/28). The University's research ethics committees are registered with the National Health Research Ethics Committee in South Africa. We explained the study aim and objectives to the potential participants and asked them to sign a consent form before their participation in the interview sessions. We also used codes to report the findings of the study and safely stored the data to ensure that the informants were kept anonymous and their information confidential. While conducting the document review, we followed the relevant standards of utility, usefulness, feasibility, propriety, accuracy, and accountability as outlined by Pawson et al. [42]. The study findings will be shared with the HAST Directorate in a feedback meeting.

Findings

In this section, we present the findings of the realist evaluation interviews with the key informant interviews combined with information from the document review. We first present the main findings in terms of Outcomes, Context, and Mechanism, and in a second part show how we proceeded to identify the CMO configurations.

Outcomes

We classified outcome themes under immediate, intermediate and long-term (**Table 4**).

Immediate outcome: decreased workload for operational staff. One of the immediate outcomes of the adherence club intervention identified as a theme from the data is related to the decrease in the workload of the operational staff (clinicians and pharmacists). This point is explained in the adherence club toolkit and by two informants:

> "[The adherence club intervention] reduces [the] patient load in mainstream care, increases available capacity for clinicians to initiate new patients on ART, and manage clinically unstable patients and patients at risk of failing ART. It can also reduce the pharmacy load

Table 4. Classification of outcomes.

Immediate Outcomes	Intermediate Outcomes	Long-term Outcomes
Decreased workload for operational staff	Decongestion of clinic	Programme standardisation
Decreased patient opportunity cost	Improved patient self-management	Retention in care and adherence to medication
		Healthier communities

doi:10.1371/journal.pone.0161790.t004

by utilising the central dispensing service for pre-packing." [DR_1]

"So it helped two things. One, for systems to be improved in clinics regarding Triage, and, secondly, getting these patients out faster and reducing waiting times, number one, and number two reducing, hmm the burden on Pharmacy." [KII_3]

"Clinicians are going to see fewer patients, there is going to be less disruption, they are going to know who is coming in, and they are going to know what their [patients] specific needs are. . ." [KII_5]

Immediate outcome: decrease in opportunity cost. Opportunity cost–the loss of other alternatives when one is chosen–in this context relates to patients losing out on either their work opportunities or family responsibilities when they spend long hours at the clinic for medication collection and routine check-up. This opportunity cost is reduced when they can collect their medication and consult quickly so that they can catch up with other activities. For the patients in the ART clubs, decreased opportunity cost was identified as an immediate outcome. The adherence club intervention is aimed at reducing the number of clinic appointments for patients. This reduces the time patients spend at the clinic as well as the frequency of the clinic attendance of the patients and, thus reduces the opportunity cost on the part of the patients. This is explained in the following excerpts:

"The adherence club only takes 45 minutes and then it is done. People can still go to work and go on with their lives. Before the adherence club, you can go to the clinic at seven in the morning, and when you leave, it is time for dinner." [DR_7]

"So, from the patient's side, it cuts down on opportunity cost, the time spent in the clinic where you are not working, you are not caring for your kids, you are not doing the things that you need to do to produce an income and look after your family." [KII_1]

The two excerpts above explain the cost and time-saving benefits to the patients. According to the participants, the adherence club intervention also offers cost and time benefits to the health system. This is echoed in the excerpt below:

"Not only does the project [adherence club intervention] reduce the patient load in mainstream care, enabling clinicians to better concentrate on new and relapsed patients, it also saves the health system and patients' invaluable time and money." [DR_3]

Intermediate outcome: decongestion of the clinic. The intermediate outcomes of the adherence club intervention that were identified by the respondents include clinic decongestion and self-management of patients. To some of the study participants, decongesting the clinic is the primary goal of the adherence club intervention. These are the words of one of the informants: "*Decongest. That was number one. . .Firstly, to decongest the clinic because clinics are really full. We have got very many patients that are on ARV's and that need follow-up*." [KII_5] This outcome was perceived as one of the main objectives and used as a selling point to the managers. One of the programme managers reported that they would say to the facility managers and clinicians, "*Look, our intention is to decongest your facility and allow more people to come in*." [KII_4] Other informants echoed this point:

"So, usually when you go to a clinic and get buy-in from the staff, you focus on the benefit of the staff, which is the decongestion and the less work, but when I sell it to patients, then I

am selling what helps the patients." [KII_1]

"So, on the other side, the benefit and health department is that they remove or decongest their clinics of these stable patients so that their resources are better utilised for new patients and patients that are struggling or unstable." [KII_8]

Intermediate outcome: improved patient self-management. A second intermediate outcome of the adherence club intervention that was identified is the facilitation of self-management in the patients. This is explained in a document as follows: "*Community-based ART helps build patient self-efficacy and the social networks that encourage patient autonomy within a supportive environment*." [DR_{12}] An informant further explained how this comes about:

"Normally, the clinic manages the patient. If the patient fails to attend his/her clinic appointment, they get contacted and are educated on how to manage their condition as well as navigate the health facility for the easy management of their disease. While in clubs, and especially in community clubs, the patients self-manage their disease because there are no clinicians to give instructions. Thus, the patients can tell when they are well and to continue taking their medication and when they are unwell to go to the clinic. [KII_8]

Long-term outcome: programme standardisation. The adherence club intervention offers a standardised ART treatment protocol. This is an important outcome for the overall ART programme. A respondent revealed what happened before the standardisation process and describes the outcomes related to the standardisation brought by the adherence club intervention.

"We know they are stable patients, but some were getting one month, some were getting two months, some were even getting more. Some were getting, maybe against what the rules were. When I say 'rules', I am talking about pharmacy practice and maybe clinical practice, but there were no guidelines. So in this way, I felt it [Adherence club] was a formalised process whereby facilities were being given guidance on how to manage these stable patients by letting go a little bit, by giving two months' supply, but also still hanging onto that ART programme that was quite very tight. . . by calling them back to the clubs." [KII_4]

Two key informants indicated the advantages of the standardisation outcome when they described how the adherence club programme offers a structured care delivery process:

"So, it [adherence club] is structured, and the rest of them. So, I'm managing the Clubs, but the other staff can comfortably know that there are 30 or 60 patients less that they are going to have to see because they are in a different setup, they have got a support group or whatever." [KII_5]

"And then, in terms of adherence from the clinical aspect, I think what, what the club offers is structure. So, everybody's scripts are aligned, everybody's blood visit is aligned, and everybody's annual clinic visit is aligned. So, by having that structure, we are able to offer a better or more routine, or the routine clinical component and so, patients do not fall through the cracks. Because we know that that does happen where they due for a test, they come to the clinic, 'someone does not review their file correctly.' [KII_{12}]

Long-term outcome: retention in care and adherence to medication. Retention in care and adherence to medication are considered the main long-term outcomes of the adherence club intervention. This is captured in these phrases:

> "So, for the patients, it was about having them being adherent, not only to their medication but to their appointments." [KII_5]
>
> "To be honest, I think for me, the main benefit is that we know that a patient came, and he has his medication." [KII_{11}]

Long-term outcome: healthier communities. Some of the study participants suggested that the adherence club intervention could ultimately lead to healthier communities.

> "And the other aspect around community awareness is I think it has had an impact to an extent because when we go out in the community as we generally try to engage that community as well—we would like to actually go to a community meeting and present that we would like to begin offering community clubs in this area." [KII_2]
>
> "I absolutely believe that it can for all the reasons that I have stated, the benefits for *all* the parties involved. I think that it is a great way that we can keep, that we can work towards keeping our communities healthier." [KII_{11}]

Context

Three themes attributed by the respondents to the context of the adherence club intervention emerged from the analysis. These context components could be categorised under local (micro), organisational (meso), and distal (macro) context components (Table 5).

Local (micro) context: availability of conducive space. Themes identified from the interviews suggested that the availability of a conducive space for conducting the adherence club activities is an important contextual element. According to the adherence club Toolkit document, "*The adherence club space varies from facility to facility depending on available infrastructure space*." Some of the informants identified the lack of space for the club activities as a challenge.

> "So, at one of our very big facilities which has very many patients, they have been going off site. They have been in the position that they have an NPO [Non-profit Organisation] working with them that is paying the rental for this off-site space. We have in the meantime tried to get it for free, and we were on the verge of a breakthrough when the manager just said 'no'. So, now, we are looking for a different place. They found one. They have not moved yet, but that is—so, so the adherence clubs are about space." [KII_4]

Local (micro) context: programme champions. Another contextual component identified from the data that could influence the mechanisms of the adherence club intervention is

Table 5. Classification of Context.

Local (Micro) Context	Organisational (Meso) Context	Distal (Macro) Context
Availability of conducive space	Sustained hierarchical pressure	Monitoring and evaluation
Programme champions	Human resources (staffing dynamics)	Higher level support
Oppressive Surveillance	Implementation methodology	Stakeholder collaboration

doi:10.1371/journal.pone.0161790.t005

'programme championship', whereby an individual is identified to 'champion' or 'lead' of the project. An informant reported that "*they* [facility managers] *identify mentors from the facility to drive or champion the process [adherence club intervention]*." [KII_4] Another informant explains further that "*Often times, the Unit manager is somebody that is really invested and would want that* [implementation of the clubs], *so that is an easy person to get things going, but the Family Physician is also an important person*." [KII_5] Informants described the importance and attributes of a champion in the adherence club programme.

> "I think it is important, very important to have someone driving it, to have someone having the oversight over, to ensure that all the bits take place. You know the Clubs Manager or the Clubs champion does not have to be doing everything. In fact, they should not be doing everything. They should be able to support or capacitate the team to actually do it. And it is necessary because we are finding that, as our programme grows more, more and more patients are joining clubs, and facilities have to do a significant amount of planning and coordinating in order to implement clubs well, and to make sure they do not fall off the bandwagon because of the size." [KII_8]
>
> "Well, it is critical because that is the person who swings it. And I mean, we have got example after example of any of the facilities that have done fantastically, you can name a person. Sometimes, you are actually very lucky, and it could even be two people or one person who was replaced by another person who carried it on. You know, we have also seen a key person leave, and this amazing programme falls apart. So, that champion is critical. [KII_{12}]

Local (micro) context: oppressive surveillance. A perception of oppressive surveillance in the form of external pressure and control was identified from the interviews as one of the aspects that influence the mechanisms that drive retention in care and adherence to medication within the adherence club programme. This denotes the practice of patients being visited at their home to ensure that they are living in supportive conditions. Oppressive surveillance is also perceived in the frequent adherence monitoring through pill counting to ascertain their adherence to medication. This also includes the increase in the number of times that they have to see the clinicians for their clinical visits

> "So when they, when clinicians enrol patients in the clubs we make sure they are assessed at specific levels before they allowed. . .and then the other issue is that they have to then see clinicians six monthly not annually because its seen as the need would be six monthly. So those patients will have extra visits." [KII_8]
>
> "So I would really love to see whether it is [home visits] adding any value because it is another huge administrative and supervisory intervention which I do not feel the facilities are really able to do and the NPO's do not seem to be doing a great job on that either." [KII_{10}]

Organisational (meso) context: sustained hierarchical pressure. While most participants suggested that the higher structures provided a supportive environment for the implementation of the adherence club, others thought the supervisors at the sub-structures were exerting pressure, especially on the facilities. This pressure was perceived through the close monitoring of the programme.

> "I think because we are watching it so closely on all levels, it is watched closely. So, if we see that [the number of clubs] are not increasing, we kind of need to, we do jump in, and we do something." [KII_5]

"Hmm, then from 2014, so two years ago was the first time I started to realise, but by then, I had already done some analysis. The data was not as good, but I had done some analysis and worked out that actually, we were not decongesting. We were just [ameliorating] the growth. So, the growth was going to be slower in the clinics, but actually, we had not managed to decongest. So, I then set targets, enrolment targets to say 'if you enrol this many persons a month into clubs then you will be in a steady state and if you enrol more than you will decongest." And that made quite a difference. [KII_{12}]

According to the adherence club toolkit, "*where a sub-district rolls out clubs as part of their ART service, it should set a target for the number of its total ART cohort that it aims to enrol in the club model. It will require two indicators to track the achievement of this target and the quality of the model: (a) number of patients enrolled in clubs in the facility (b) number of patients retained in the facility clubs."* Some participants suggested that, because they are given targets to meet in terms of the number of patients to be admitted into the clubs, the number of clubs to be created within a period, and the percentage of ART patients to be in clubs, they were being pressured:

"So, I then set targets, enrolment targets to say 'if you enrol this many persons a month into clubs, then you will be in a steady state and if you enrol more, then you will decongest." And that made quite a difference. We had this flurry of interest because it was the first time anyone had been given any kind of target of where they should be trying to go. The first six months, we had an *amazing* response and then for the next year or so, we had quite a few facilities that were really struggling with that rapid growth and then, you know, they were really just struggling with the logistics of clubs." [KII_{12}]

Organisational (meso) context: staffing dynamics. Respondents identified human resource availability and other staffing dynamics as important contextual components that impact on the implementation of the adherence club intervention. According to the adherence club toolkit document, five (cadres of) staff are needed to successfully run the intervention in a facility–a club manager, club nurse, clinician, counsellor, and pharmacist or assistant pharmacist. A document describing the adherence club intervention states that the intervention "*requires sufficient human resources to support and run the clubs and cannot [should not] drain current facility staffing as the clubs expand.*" [DR_3]

An informant suggested that when the facility does not have all the required cadre for running the adherence club, some level of flexibility should be considered. This idea of adaptability is explained by two informants:

"So, the facility did not have all those cadres of workers available. Sometimes, the same nurse had to be the club manager and also had to be the club PN [Professional Nurse] and also had to do different roles, but with one person. . . So, in that way, although the outline of how a club should run had all the definitions of what these people should be doing and who they should be, we kind of still, kind of tailored it to the context." [KII_4]

"We try to encourage all the nurses in facilities to actually be a Club nurse. So, you could rotate through that role and because then you see the value that all those patients, even though they not in your waiting room anymore, they are still your patients you know." [KII_9]

The various staff adaptations provide different staffing contexts for the implementation of the adherence club intervention within different facilities.

Organisational (meso) context: implementation methodology. Some of the participants identified that the use of a particular method for the implementation of the adherence club intervention within the facilities had an influence on the results that they obtained. This identified the implementation methodology as an important organisational contextual factor for the successful implementation of the adherence club intervention

"It is a breakthrough series collaborative model. If you work collaboratively over time which is a series thing and it is quite structured, then you will get breakthrough results. First of all, it is very important that it has a very clear aim and that aim has got to do with, generally with patient outcomes. So, it has to be something that is, people feel it is worth doing. So, even though we might have said our aim was to spread the clubs to you know all the ARV clinics in the City we all knew that that aim was, actually, to improve retention in care and to improve initiation, early initiation across the City *by*, our change was the clubs, by spreading the clubs but we all had in mind that this was going to have great patient outcomes, that was the intention. So to spread the clubs and get worse patient outcomes would not have been a desirable outcome." [KII_4]

"Yes, that strategy [breakthrough series collaborative model] was very effective. We are actually using it with Province in another model now where we identify high burdened facilities. We ask them to identify their club teams, they come for a training; we do a training; the Mentors that we have identified to offer support during implementation; they are there for support; follow them for 6 months; we return again; we check in how it is going; we address any key challenges; we set new goals and we go." [KII_7]

Distal (macro) context: emphasis on monitoring and evaluation. Other contextual factors that respondents reported as important for the successful implementation of the intervention were monitoring and evaluation services in the programme:

"So, monitoring and evaluation become a big thing for you in clubs, because it is pointless you are putting patients there [in the adherence club], but you cannot determine if you are retaining them in care. And what I also would like to point out is that besides these patients remaining in care, they have got one clinical visit per year. Only one that have contact with the clinician." [KII_8]

"It [implementing adherence clubs] does require planning and support and monitoring and evaluation, all of those bits are important. And so, when you see other provinces trying to do it, you do actually want to say "you have got to have some structures in place." [KII_2]

"So, there is a monthly meeting where they look at the ARV data. And when I set those targets, the sub-district manager jumped on them and put it into the programme for implementation. . ." [KII_{12}]

Distal (macro) context: higher level support. Another theme that emanated from the data relating to the context is the support provided to facilities from higher structures or departments. We found that during the implementation phase, the steering committee provided most of the support. This support is explained by a participant:

"We train them on the model, and they would come up with a plan for how they were going to roll it out. And they would then be allocated a club mentor, which was either one of the HAST MOs [Medical officers] or somebody else. . . And so, that means that that person,

once they have been trained and went back, you would pop into the facility and answer questions and support where they were struggling, etcetera, for a given amount of time. And then, six months later, we [steering committee] would have another learning session where all those teams would come back and report on where they were [with club implementation], what they were struggling with, etcetera and get input. And then, we did that another time. So, it was over three sessions. And those mentors for the facilities were supposed to kind of, once the facilities were up and running, step back and then move to another facility. They did that in some places, in some places they still provide some mentorship. That is the one set of training that took place." [KII_{10}]

The importance of on-going support is expressed by another participant:

"So, I think the advantage also is District's presence . . .. district support . . . I think that's important. . . .So, the advantage of that was to get buy-in from everybody, and we did, it was actually a success. . .." [KII_3]

One other informant made the comparison between facilities that received support for the running of their adherence clubs, to those that did not, thus highlighting the important role that on-going support plays in propagating better retention in care and adherence outcomes.

"So, that is why Ubuntu was one of the most successful. Like I say, they received support from MSF, you know, during the implementation of these clubs. It was successful . . . So, it is about what support did MSF give a clinic initially." [KII_8]

Distal (macro) context: stakeholder collaboration. Stakeholder collaboration was identified as an important contextual theme for the implementation of the adherence club intervention. For instance, the programme's steering committee, consisting of the various stakeholders and the Western Cape HAST Directorate, provided the political clout for the implementation of the adherence club intervention within the Cape Metropole. Three informants highlight the role and importance of stakeholder collaboration:

"At the provincial level, we [MSF] still sit as a technical partner on the steering committee, and that is where I sit with a member of Institute for Care Improvement and others and HAST MOs [Medical Officers], etcetera and Pharmacists and data capture clerks, all of that. And that steering committee, I think, is vital to be able to continue to engage with the change in dynamics of implementing the programme." [KII_{12}]

"So, that is me and the HAST managers, the HAST MOs [Medical Officers], the leader of the HAST team at Province, the Pharmacist. We all meet once every two months and go through problems or things that we are changing in the club system or, for instance, when we finalised the SOP or now as we want to roll out family clubs. We all sit together and make a plan." [KII_1]

This collaboration between stakeholders can lead to interactions and possible programme buy-in. The role of collaboration is expressed in these excerpts.

"Okay, and that is the model that we used in that project. And because I had done that project, and it was done with the municipality with City Health, and the Head of the City HAST had been working for . . . at the time, and it was a collaboration, so they were also involved

in our project, and she then moved across to the City." [KII_4]

"Well, the [HAST] Directorate gave us the space to be able to make that collaborative effort to work in those clinics and so both the City and the Province had to work together and both of them had to agree that we could do this collaboratively." [KII_2]

Mechanisms

We identified the following potential mechanisms during our analysis. We classified the mechanisms according to those operating at the management/provider level and the mechanisms at the patient level (Table 6).

Providers/managers mechanism: buy-in. Buy-in from stakeholders such as facility managers, operational staff, and patients is central to the successful rollout, implementation, and execution of the adherence club intervention. The adherence club toolkit emphasises the importance of buy-in from the facility managers and suggests that the facility managers take 'ownership' of the intervention. According to a document reviewed, if the facility manager and the clinicians buy into the intervention, then they could "*maintain good long-term adherence in patients on ART treatment by creating an environment within the health facility for more convenient clinical visits that is conducive to the patients' lifestyle needs.*" [DR_9]

Most of the participants identified buy-in as an important driver of the possible success of the adherence club intervention. This is expressed in the words of these key informants:

"I realised to get this programme working right; you need everybody's buy-in from the beginning. . ." [KII_{11}]

"So, I think at times it is the degree, I guess, I call it of buy-in from the Facility Manager, the knowledge of the Facility Manager around ARV's and the needs of the various people in the facility, whether it is the staff, the patients, or whoever." [KII_1]

Providers/managers mechanism: interaction. The concept of interaction as a mechanism of action for implementation of the adherence clubs flows from the collaborations that exist between the various stakeholders. According to a document written by MSF, "*The extent to which patient groups engage in mutual support will probably be context- specific and should be community/patient driven. As a minimum, community stakeholders should be involved in planning and implement community-based models.*" An excerpt from the interviews affirms this point of view:

"So, because of the earlier work we had done, we all had relationships, we all had, a lot of people in the ARV [ART programme], because it was ARV [the ART programme], so a lot

Table 6. Classification of Mechanisms of action.

Provider/Management Level	Patient-level
Buy-in	Motivation
Interaction	Group dynamics—Social/Peer support
Motivation	Nudging
Problem-solving	Encouragement
	Trust
	Bonding
	Learning
	Fear

doi:10.1371/journal.pone.0161790.t006

of people in both Province and in the City had an experience of this methodology [Breakthrough Series Collaborative Model] and we had won a Mpumelelo award with it so we were all confident that it was good methodology. We had fantastic results, we all enjoyed working like that. It was very collaborative; very creative." [KII_8]

Providers/managers mechanism: motivation. Motivation on the side of the clinicians and counsellors working with PLWHA on ART was identified from the data as an important mechanism of action. It is related to the fact that the adherence club intervention offers a structured and somewhat predictable work schedule so that they can anticipate their workload. An informant explained this in detail:

"You can plan for it [preparations for the club meetings] proactively, "these are my clubs. These are the dates when they are going to come through the year. Let me see that my scripts have gone in two weeks before. Let me see that I have got the medicine pre-packed the day before. Let me see that the patients that didn't come, have been contacted." [KII_{10}]

"So, it is structured, and the rest of them, so I am managing the clubs, but the other staff can comfortably know that that's 30 or 60 patients less that I am going to have to see because they in a different setup, they have got a support group or whatever. So it had benefits for both parties." [KII_2]

Providers/managers mechanism: problem-solving. These interactions can also lead to effective problem-solving, which in turn increases self-efficacy and trust among the different stakeholders

"There still is a need for the steering committee' because that committee is basically doing the technical work and basically a lot of the focus is now on innovation because we have rolled out clubs in a particular way. . . MSF did it in a particular way, in a very well-resourced way, but, you know, the reality out there is that we don't have all those cadres." [KII_7]

Patient level mechanism: motivation. Motivation is one of the concepts identified as a possible mechanism through which the adherence club intervention works. Motivation in itself is achieved in more than one way. First, the motivation of patients on ART could be achieved through the trusting relationship between the club facilitators and the patients. This trusting relationship would inspire the patients to take to heart what they have learned in the peer education, counselling or consultative sessions. This is reflected by an informant who explained the importance of the counsellors acquiring the skills to develop trust relationships with their patients:

"Is there basic information that we can translate to a lay worker to say 'this is what you do to make someone feel comfortable, this is how you build trust, this is how you do not build trust.' I think that are key skills that they need [without which], I don't think the adherence clubs are going to work." [KII_{10}]

Another source of motivation and positive enforcement that may influence the patients to be retained in care is provided through the continuity of care that the patients get from the

Club Facilitators and the clinicians that see them on a regular basis. This point is explained by an informant:

> "Some of the patients value the other patients being there, but some of them value the actual facilitator who is someone that they get to know. At least, they see the same person every time, whereas, normally, if they go to the clinic, they will see a different clinician every time." [KII_1]

Continuity of care offers the patients the chance to familiarise themselves with the same Club Facilitator and Club Nurse. This is supposed to instil trust in the patients, which motivates them to attend their club appointments, especially when the Club facilitators get to know the patients personally and call them by name. This is believed to motivate the patients to be retained in care and adhere to their medication.

Patient level mechanism: group dynamics—social/peer support. Grouping patients together for their ART treatment is not only about providing them with easy access to medication but about the relationships formed between the group members. Most of the interview participants identified some interaction among the group members to have a positive impact on retention in care and adherence. It is suggested in a document that "*the relationship between providing social support and improved adherence to treatment is well established*." [DR_{12}] The concept of group dynamics emerged in the document analysis:

> "Club members establish a positive group dynamic over time, which renders much-needed peer support for adherence to lifelong treatment." [DR_2]
>
> "[The adherence club] creates [an] opportunity for establishing group dynamic and peer support." [DR_1]

These two quotes identify peer support as a product of the group dynamics within ART clubs. The clubs are thought to offer social support that could foster retention in care among the group members and ultimately, medication adherence "*The relationship between providing social support and improved adherence to treatment is well established*." [DR_{12}] This theme of positive group dynamics also emerged from the interviews. One of the informants reported that when the group members convene from time to time, they familiarise themselves with each other, which creates a positive atmosphere for better relationships and sharing:

> "See the same people every two months. They get to know each other a little bit, which also helps build a little bit of *camaraderie* and understanding that there are other people in the same situation, and they allow sharing between people." [KII_1]

Medical anthropologists describe this concept as 'biosociality'- a relationship formed when patients collectively share meanings of the extreme experiences of illness and stigmatisation causing a sense of bonding among the patients [43]. Two participants explained further that:

> "It is about being in a group of patients who have similar challenges that I have. Number one, we are all HIV positive, and we all need to be put on treatment. So that just kind of breeds some loyalty, and so it also gives you an idea as to what the health status is having this 'dreaded disease' as people called it back in the day." [KII_5]
>
> "The adherence club model gives a bit of social fabric where people come together in a group, in a support group structure and actually get to engage with other people just like them, maybe not from the same exact suburb but certainly the same community of people living with HIV." [KII_7]

Another participant explained that being managed as a group brings about a shared identity which leads to a peer supportive environment: "*It is about gaining the sort of peer support environment where we do not feel as alienated as being the only person with HIV and able to talk through things and to get your treatments quickly.*" [KII_1] Key informant 5 explains in detail how the group identity develops:

> "But you look around you, and you can see "But this person, you know, has the same thing as I, but he or she is looking, is healthier than I", and so you can converse and, "What is happening? How come you look like this and I look like that, and we have the same issues?" "Hmm, you know it is kind of. . ." They share their lives with each other and you know there is an old saying that says "a burden shared is a burden halved." [KII_5]

Patient level mechanism: nudging. Another salient mechanism that was identified by interviewees is nudging—helping people to make the right choices by setting default options in a specific way. The standards of practice of the adherence club have clauses that oblige patients to attend club activities. A method applied by the operational staff is to 'nudge' the patients by reminding them that if they do not adhere to the club activities and collect their medication within the 5-day grace period, they would be returned to the main clinic care (which has the challenges of long waiting times to see a clinician and to collect medication at the clinic pharmacy). This is captured in the following excerpts:

> "Patients can be removed from club care and returned to mainstream care where more intensive clinical or adherence follow-up is required. A patient exits the club when he/she misses a mandatory club session and fails to attend the clinic within five days. Patients determined by the club nurse to require more regular follow-up and those with elevated viral loads are also returned to mainstream care." [KII_8]
>
> "We decide our grace period, but we are also going to be stricter with our club patients to stop them coming each time after the club session. They will need to see the Clubs Manager when they are late." [KII_1]

Patient level mechanism: encouragement. According to the study participants, treating patients in a group is considered to foster collaborations and 'friendship' among the group members. In the group-based setting, the members tend to encourage and motivate one another towards adhering to their medication and attending their clinic appointments. This concept is further explored by another key informant:

> "So, for me, it [adherence club intervention] would be a means of making that connection with the people in your group, and you see each other every two months. You are going to see each other every two months and so you would want to see the person. You would want to know, you know, 'They were looking so badly, I wonder what is happening' and hopefully, they connected and swapped numbers and kind of messaged each other as a form of encouragement." [KII_{10}]

Patient level mechanism: trust. Trust was also identified as an important mechanism through which the adherence club intervention would achieve its objectives. One of the interviewees explained that patients could trust the system if the clinic or operational staff could deliver the services as promised to the patients:

"So, I think that in a sense, when you have given what you said you are going to give is an incentive, and we have definitely seen in facilities that struggle to have this system in place that the patients are far less willing to be on time or come on their club day. So, I think that does have an, have an impact." [KII_{12}]

Patient level mechanism: bonding. Bonding among the patients grouped together was identified as another mechanism of action to promote retention in care and adherence to medication among patients on ART. According to the Standard operating practice document,

"The Club environment needs to provide a space that is conducive for people in the club to chat to each other, as part of the aim of the club system is to *foster bonding* between patients so that they can support each other in the ongoing need for adherence." [DR_9]

A participant explained that bonding can also be achieved in the group when patients come together in the club session:

"But you know, from my observations, people enjoy coming and meeting others. They build relationships maybe with unlikely people that they would otherwise not have met. And I have definitely seen some club members who disclosed, you know, social or personal issues in that space because they feel like they can trust this space." [KII_7]

Patient level mechanism: mutual learning. Another mechanism that was identified to be activated in the context of the adherence club implementation is mutual learning. According to some of the interviewees, patients learn from one another while in the group, and this can help them to self-manage their disease easily.

"So, you learn from each other within the group. You learn about, you know, the adherence to medication. You are encouraged to come to your appointments. You are encouraged to disclose, you encouraged to live your life a little bit, you know, with positivity and not with negativity and think that you know 'I am dying tomorrow.' So, there is a lot to be gained from being in an adherence club, because for me, an adherence club is a form of a support group you know. You see the same people every two months, you visit, and you can chat, and you hear what their life is all about and you can, you can kind of do the same thing as it were." [KII_9]

Patient level mechanism: fear (of losing the benefits of the club). Fear was identified from the data as one of the driving forces behind their faithful attendance of the club activities. This fear is mostly associated with losing the benefits the patients feel that the club has over the standard clinic care.

"You know, patients do not want to leave the club because, if you have a job, you know, they come get their medicine, in and out but if you just have to go home to do the washing, whatever, and you got a little bit more time then you will lose the interaction, I think. So it depends on where you are in your life, I think." [KII_{10}]

Discussion

Identified CMO configurations

Using the configurational analysis approach, our goal at the second stage of analysis was to consider the possible interactions between each of the specific components and identification

of context, mechanism, and outcome. We applied Berman's policy implementation model to illustrate how various CMOs occur at each level [44]. According to Berman's model, policy implementation follows the following processes: (1) translation of the policy to a programme; (2) programme adoption by managers; (3) programme implementation by providers, and (4) programme uptake by patients. Our discussions here focus on the last three processes. We identified CMO configurations for three groups of actors: the providers, the managers, and the patients.

Provider perspective. Providers will likely adopt and implement the adherence club programme if the policy environment is conducive and if it suits their interest. The political clout of the programme contributes to its spread and implementation. The informants suggest that this is achieved through the steering committee, district health support, collaborations between the facilities and the government health organisations. This results in buy-in from the facility's managers and providers, and the feeling of soft coercion by the facility managers. The stakeholder collaboration also provides an atmosphere of effective problem-solving and empowerment, consequently contributing to the successful adoption and implementation of the programme. Support in the form of training and guidance provided by the steering committee to facilities also facilitates uptake by providers. These mechanisms are assumed to promote the successful rollout to the adherence club intervention in various primary health care facilities. The contingent causality explanation of this perspective is represented in below.

Management perspective. The CMO for the managers focuses on how the programme is supposed to work at the level of the clinical management. Clinic managers are more likely to adopt and implement the strategy if their buy-in and motivation are achieved through support from the district level, collaborations with non-governmental organisations and the central drug dispensing unit. Their motivation may be increased by the prospect that the programme would decongest the facility and lighten the load of the operational staff. They can be coerced to adopt the programme through hierarchical pressure and standard setting. If the right balance is struck, successful implementation and adaptation of the intervention become more likely, and its potential to standardise the ART programme, decrease the workload and decongest the clinic higher. describes the CMO configuration at this level.

Patient perspective. Two CMO configurations emerged related to the patient perspective, suggesting different pathways through which the outcomes could be achieved. The first CMO, illustrated in, represents the most popular view of the designers and managers. Patients are assumed to regularly attend the adherence club and to take their drugs if the mechanisms of patient empowerment, positive group dynamics (biosociality), learning, trust, motivation, and social support are triggered. This requires the buy-in from the facility manager and operational staff, the availability of resources such as operational staff, patient selection criteria, a conducive meeting space, and the contributions of other organisations towards running the adherence club programme as context factors (context). When the ART club is tailored to its 'target' group of patients and its context and as such triggers the mechanisms, it is assumed to encourage patients to self-manage their disease, be retained in care, and adhere to their medication.

An alternative CMO indicates that patients may attend the adherence club out of fear to have to go back to the main clinic care if they fail to attend their club sessions. Within different contexts, different mechanisms are activated, for instance, patients may either feel motivated by the contextual conditions or be nudged. Mechanisms that were identified to be triggered by the context of this situation included fear of returning to the main clinic and losing the benefits of the clubs, feeling nudged by the patient surveillance activities (attendance registration, pill counting for adherence monitoring), and being coerced by club rules and processes. These could plausibly contribute to behaviours of increased retention in care and adherence to medication among the patients.

Framing the CMOs in existing theory

There are many approaches to initiating change in health behaviour, from external pressure and control to the use of incentives or rewards. The first patient-related CMO configuration typifies the use of incentives and rewards and identifies motivation as an important mechanism of action which prompts patients to attend the adherence club sessions, based on what the adherence club promises to offer. With motivation as a central ingredient, this CMO could be linked to the self-determination theory, according to which motivation–"the inherent tendency to seek out novelty and challenges, to extend and exercise one's capacities, to explore, and to learn" [45] plays a central role in determining the actions of the individual (in this case retention in care and adherence to ART medication). According to Ryan and Deci [45], motivation highlights the use of evolved inner resources for personality development and behavioural self-regulation. Thus, treatment environments that afford autonomy and support confidence are likely to enhance retention in care, adherence and consequently, positive health outcomes [45].

The second patient-related CMO, suggests that the patients are being nudged, pressured, or controlled to attend club activities. In other words, an external force prompts the patients to attend the club activities. Nudging theory suggests that humans are prone to making bad decisions because they are easily tempted to, and that they should be helped to make better choices. According to Thaler and Sustein, gently helping people to make better choices by "setting default options, and other similar seemingly trivial menu-changing strategies, can have huge effects on outcomes" [46]. The effects of well-chosen default options provide just one illustration of the gentle power of nudges. The choice architects (clinicians) are supposed to rearrange the physical and social environment in order to make people change behaviour to make better choices [46]. Nudging could also be implemented through the threats of losing the benefits of the adherence club benefits when the patients fail to attend the adherence club activities.

Elements of an initial programme theory of the adherence club programme

The implementation of the adherence club intervention occurs in a policy environment driven by goals of decongesting the health system and achieving better retention in care and adherence rates among patients on ART. The HAST Directorate at the provincial level oversees the implementation and running of the programme. This is done in part by a steering committee with representatives from HAST, MSF, TAC, and the City of Cape Town. The role of the steering committee is to monitor the implementation of the intervention and engage with the changes in the dynamics of implementing the programme to come up with solutions to address surging challenges. From the steering committee, decisions on how to improve the roll-out and implementation of the adherence club intervention at the facility level are taken and shared with the sub-district HAST offices that micromanage the implementation process at the various primary health care facilities. The decisions are then adopted by the ART unit of the facility, and the clubs are run accordingly. Monitoring and evaluation of the performance of the adherence club intervention at the facility levels inform the decisions taken at the level of the steering committee.

The different provisional CMO configurations for different actors at various levels show how each level is related to the others and how the effective adoption and implementation needs to be secured at the different levels of the health system, from the central policymaking and programme level, through the district and sub-district to the facility-level. Combining the provider's perspective, the management's perspective and the patient's perspective of how the adherence club programme works, indeed, provides an initial overarching programme theory of the adherence club programme.

From a realist perspective, in a multilevel system such as the health system, the outcome patterns (proximal, distal, negative, intended and unintended) of one level may constitute the proximal context in the next level of the system [47]. For instance, decisions made on the number of adherence clubs to be implemented within a timeframe at the sub-district level might become a contextual component within which it is implemented at the facility management level and consequently the execution of the programme. In a similar manner, the outcomes at the ART unit and facility management could form part of a distal context within which the programme is being implemented. Understanding how actors and their decisions operating at these different levels interact and influence each other can provide a more comprehensive understanding of the adherence club intervention at the final level of implementation and execution.

Rigour and Trustworthiness

The rigour and trustworthiness of the study findings were improved by applying the following principles. We conducted a pilot of the interview guide, which helped us to assess the type of information that the questions we asked were likely to produce. We applied the processes of method and source triangulation. The document analysis allowed us to triangulate the information from the interviews. In addition, a wide range of stakeholders was recruited as study participants. We conducted iterative questioning in data collection dialogues with the study participants. We conducted member checks with the study participants of the theories that were formulated if they reflected their views. In conventional qualitative studies, researchers are required to apply the principle of bracketing (neutral territory) during the entire study by which we acknowledged and side-lined our preconceptions before engaging in the study [48], on the contrary, conducting a realist interview requires the investigators to engage with the respondents [21]. Conducting a document review provided us with relevant concepts and theories to engage with the study participants. In this process, we took neither an insider nor an outsider perspective about the programme [21]. Trustworthiness was enforced by actively searching for disconfirming evidence through negative case analysis [49] or deviant case analysis [26] and by keeping an audit trail. Finally, we followed the relevant aspects of the Criteria for Reporting Qualitative Research (COREQ) outlined by Tong, Sainsbury, and Craig [50].

Study Limitations

We applied the process of iterative consultation with the stakeholders during the configurational analysis process. We do acknowledge that this process has the potential to introduce confirmation bias, whereby, with subsequent consultations, the stakeholders tend to agree with whatever the evaluators have presented. We limited this bias by asking the stakeholders to think differently or in a way to improve the theories. Another limitation of the study is related to the fact that while obtaining the assumptions of the programme designers and managers, we did not include stakeholders such as facility managers, the operational staff and patients. Consequently, the programme users' perspectives were obtained on the basis of interviews with managers and providers rather than the programme users.

Conclusion

Based on the interviews with the adherence club programme designers and managers and a document review, three perspectives of how the adherence club intervention is expected to be successfully implemented and achieve the goals of patient retention in care, adherence, and clinic decongestion were identified. This represents the first step towards developing programme theories that explain how the adherence club intervention is expected to work, for

whom and under what circumstance. The next step is to assess the evidence on the various mechanisms of action working in other group-based adherence interventions and review the literature on theories that have been explored to explain retention in care and adherence to ART. This will allow us to formulate a programme theory that represents a hypothesis of how the adherence club works which we will subsequently test in empirical studies.

Supporting Information

S1 Fig.
(DOCX)

S2 Fig.
(DOCX)

S3 Fig.
(DOCX)

S4 Fig.
(DOCX)

S5 Fig.
(DOCX)

S6 Fig.
(DOCX)

S1 File. Key informant interview guide. Additional file 1.
(DOCX)

Acknowledgments

We would like to thank Ms Suhair Solomons, Mrs Neshaan Peton, Dr Ebrahim Kriel, and Dr Shahieda Jacobs for their technical contribution to the study.

Author Contributions

Conceptualization: FCM BM BVW.

Data curation: FCM.

Formal analysis: FCM BM.

Funding acquisition: FCM BVW.

Investigation: FCM BVW.

Methodology: FCM SVB BM.

Project administration: FCM.

Supervision: SVB BM BVW.

Validation: FCM SVB BM.

Visualization: FCM BM.

Writing – original draft: FCM BM.

Writing – review & editing: FCM SVB BM BVW.

References

1. UNAIDS. Number of people living with HIV; 2016. Available: http://aidsinfo.unaids.org/.
2. Uebel K, Lombard C, Joubert G, Fairall L, Bachmann M, Mollentze W, et al. Integration of HIV Care into Primary Care in South Africa: Effect on Survival of Patients Needing Antiretroviral Treatment. J Acquir Immune Defic Syndr. 2013; 63(1):e94–e100. doi: 10.1097/QAI.0b013e318291cd08
3. Treatment Action Campaign, +SECTION 27. South Africa's AIDS and TB response: Code Red? Treatment Action Campaign; 2015 p. I–57. Report No.:12. Available: http://www.nspreview.org/2015/06/10/nsp-condition-critical-code-red/.
4. Wilkinson L. ART adherence clubs: a long-term retention strategy for clinically stable patients receiving antiretroviral therapy. S Afr J HIV Med. 2013; 14(2):48–50. doi: 10.7196/SAJHIVMED.924
5. Kagee Remien, Berkman Hoffman, Campos Swartz. Structural barriers to ART adherence in Southern Africa: Challenges and potential ways forward. Glob Public Health. 2011; 6(1):83–97. doi: 10.1080/17441691003796387 PMID: 20509066
6. Kheswa J. Non–Adherence to Antiretroviral Treatment by People Living with HIV/AIDS in Black Communities in South Africa: Socio-Cultural Challenges. Mediterr J Soc Sci. 2014; 5(14):450–457. doi: 10.5901/mjss.2014.v5n14p450
7. Adefolalu A, Nkosi Z. The Complex Nature of Adherence in the Management of HIV/AIDS as a Chronic Medical Condition. Diseases. 2013; 1(1):18–35. doi: 10.3390/diseases1010018
8. Roura M, Busza J, Wringe A, Mbata D, Urassa M, Zaba B. Barriers to Sustaining Antiretroviral Treatment in Kisesa, Tanzania: A Follow-Up Study to Understand Attrition from the Antiretroviral Program. AIDS Patient Care STDS. 2009; 23(3):203–210. doi: 10.1089/apc.2008.0129 PMID: 19866538
9. Bezabhe W, Chalmers L, Bereznicki L, Peterson G, Bimirew M, Kassie D. Barriers and Facilitators of Adherence to Antiretroviral Drug Therapy and Retention in Care among Adult HIV-Positive Patients: A Qualitative Study from Ethiopia. PLoS ONE. 2014; 9(5). doi: 10.1371/journal.pone.0097353
10. Ford N, Mills E. Simplified ART delivery models are needed for the next phase of scale-up. PLoS Med. 2011; 8(7):e1001060. doi: 10.1371/journal.pmed.1001060 PMID: 21811405
11. Bemelmans M, Baert S, Goemaere E, Wilkinson L, Vandendyck M, Cutsem G, et al. Community supported models of care for people on HIV treatment in sub-Saharan Africa. Trop Med Int Health. 2014; 19(8):968–77. doi: 10.1111/tmi.12332 PMID: 24889337
12. Decroo T, Rasschaert F, Telfer B, Remartinez D, Laga M, Ford N. Community-based antiretroviral therapy programs can overcome barriers to retention of patients and decongest health services in sub-Saharan Africa: a systematic review. Int Health. 2013; 5(3):169–79. doi: 10.1093/inthealth/iht016 PMID: 24030268
13. Campion E. Treating Millions for HIV—The Adherence Clubs of Khayelitsha. The N Engl J Med. 2015; 372(4). doi: 10.1056/NEJMp1414213
14. Vandendyck M, Motsamai M, Mubanga M. Community-based ART resulted in excellent retention and can leverage community empowerment in rural Lesotho, a mixed method study. HIV/AIDS Res Treat Open J. 2015; 2(2):44–50. doi: 10.17140/HARTOJ-2-107
15. Rasschaert F, Telfer B, Lessitala F, Decroo T, Remartinez D, Biot M, et al. A Qualitative Assessment of a Community Antiretroviral Therapy Group Model in Tete, Mozambique. Plos One. 2014; 9(3):e91544. doi: 10.1371/journal.pone.0091544 PMID: 24651523
16. Bateman C. MSF again paves the way with ART. S Afr Med J. 2013; 103(2):71–73. doi: 10.7196/SAMJ.6666 PMID: 23374312
17. Luque-Fernandez M, Cutsem G, Goemaere E, Hilderbrand K, Schomaker M, Mantangana N, et al. Effectiveness of Patient Adherence Groups as a Model of Care for Stable Patients on Antiretroviral Therapy in Khayelitsha, Cape Town, South Africa. PLoS ONE. 2013; 8(2). doi: 10.1371/journal.pone.0056088
18. Grimsrud A, Sharp J, Kalombo C, Bekker L-G, Myer L. Implementation of community-based adherence clubs for stable antiretroviral therapy patients in Cape Town, South Africa. J Int AIDS Soc. 2015; 18(1). doi: 10.7448/IAS.18.1.19984
19. Mukumbang FC, Van Belle S, Marchal B, Van Wyk B. A realist evaluation of the antiretroviral treatment adherence club programme in selected primary health care facilities in the metropolitan area of Western Cape Province, South Africa: A study protocol. BMJ Open. 2016; 6(4):e009977. doi: 10.1136/bmjopen-2015-009977 PMID: 27044575
20. Pawson R, Tilley N. Realistic Evaluation. 2nd ed. London: SAGE Publications; 1997.
21. Manzano A. The craft of interviewing in realist evaluation. Eval. 2016; 22(3): 342–360. doi: 10.1177/1356389016638615

22. Astbury B, Leeuw FL. Unpacking Black Boxes: Mechanisms and Theory Building in Evaluation. Am J Eval. 2010; 31(3):363–381. doi: 10.1177/1098214010371972
23. Birckmayer JD, Weiss CH. Theory-Based Evaluation in Practice: What Do We Learn? Evaluation review. 2000. Available: http://erx.sagepub.com/content/24/4/407.short. Accessed 18 Feb 2016.
24. Pawson R, Sridharan S. Theory-driven evaluation of public health programmes. In: Killoran A, Kelly M (eds) Evidence-Based Public Health: Effectiveness and Efficiency. Oxford: Oxford University Press, 43–61; 2009.
25. Bowen G. Document Analysis as a Qualitative Research Method. Qualitative Research Journal. 2009; 9(2):27–40.
26. Porter S. Validity, trustworthiness and rigour: reasserting realism in qualitative research. J Adv Nurs. 2007; 60(1):79–86. doi: 10.1111/j.1365-2648.2007.04360.x PMID: 17824942
27. Pawson R, Greenhalgh T, & Harvey G, Walshe K. Realist review–a new method of systematic review designed for complex policy interventions. J Health Serv Res Policy. 2005; 10 Suppl. 1:21–34. doi: 10.1258/1355819054308530 PMID: 16053581
28. Leeuw F. Reconstructing Program Theories: Methods Available and Problems to be Solved. Am J Eval. 2003; 24(1):5–20.
29. Blamey A, Mackenzie M. Theories of Change and Realistic Evaluation Peas in a Pod or Apples and Oranges? Eval. 2007; 13(4):439–55. doi: 10.1177/1356389007082129
30. Creswell JW. Research design: Qualitative, quantitative, and mixed methods approaches. Sage Publications; 2013.
31. O'Reilly M, Parker N. "Unsatisfactory Saturation": a critical exploration of the notion of saturated sample sizes in qualitative research. Qual Res. 2012; 13(2); 190–197. doi: 10.1177/1468794112446106
32. Pawson, R, Tilley N. Realist Evaluation. 2004; http://www.communitymatters.com.au/RE_chapter.pdf. Accessed 24 Nov 2015.
33. Wand T, White K, Patching J. Realistic evaluation of an emergency department-based mental health nurse practitioner outpatient service in Australia. Nurs Health Sci 2011; 13(2):199–206. doi: 10.1111/j.1442-2018.2011.00601.x PMID: 21595814
34. Miles MB, Huberman AM. Qualitative data analysis. 2nd ed. Thousand Oaks, California: Sage Publications; 1994.
35. Braun V, Clarke V. Using thematic analysis in psychology. Qual Res Psychol. 2006; 3(2):77–101.
36. Gerbic P, Stacey E. A purposive approach to content analysis: Designing analytical frameworks. The Internet and Higher Education. 2005; 8(1):45–59. doi: 10.1016/j.iheduc.2004.12.003
37. Linsley P, Howard D, Owen S. The construction of context-mechanisms-outcomes in realistic evaluation. Nurse Researcher. 2015; 22(3):28–34. doi: 10.7748/nr.22.3.28.e1306 PMID: 25587864
38. Pawson R, Manzano-Santaella A. A realist diagnostic workshop. Eval. 2012; 18(2):176–91. doi: 10.1177/1356389012440912
39. Wong G, Westhorp G, Pawson R, Greenhalgh T. Realist Synthesis: Rameses training materials, 2013. Available: http://www.ramesesproject.org/media/Realist_reviews_training_materials.pdf.
40. Pawson R. Evidence-Based Policy: A Realist Perspective. 1st ed. Sage Publication; 2006.
41. Westhorp G. Realist Impact Evaluation: An Introduction. A Method Lab Publication. A Method Lab Publication; 2014. Available: https://www.odi.org/sites/odi.org.uk/files/odi-assets/publications-opinion-files/9138.pdf.
42. Pawson R, Boaz A, Grayson L, Long A, Barnes C. Types and Quality of Knowledge in Social Care. Social Care Institute for Excellence, London; 2003. Available: https://www.kcl.ac.uk/sspp/departments/politicaleconomy/research/cep/pubs/papers/assets/wp18.pdf.
43. Marsland R. (Bio)Sociality and HIV in Tanzania: Finding a Living to Support a Life. Med Anthropol Q. 2012; 26(4):470–85. doi: 10.1111/maq.12002 PMID: 23361880
44. Berman P. The Study of Macro and Micro Implementation of Social Policy. Santa Monica, CA: RAND Corporation, 1978. Available: http://www.rand.org/pubs/papers/P6071.
45. Ryan RM, Deci EL. Self-determination theory and the facilitation of intrinsic motivation, social development, and well-being. American psychologist. American Psychological Association; 2000; 55(1):68. Available: http://psycnet.apa.org/journals/amp/55/1/68/. PMID: 11392867
46. Thaler RH, Sunstein CR. Nudge: Improving decisions about health, wealth, and happiness. Yale University Press: New Haven & London; 2008.
47. Pedersen L, Rieper O. Is Realist Evaluation a Realistic Approach for Complex Reforms? Eval. 2008; 14 (3):271–93. doi: 10.1177/1356389008090856

48. Tufford L, Newman P. Bracketing in Qualitative Research. Qual Soc Work. 2010; 11(1):80–96. doi: 10.1177/1473325010368316
49. Shenton AK. Strategies for ensuring trustworthiness in qualitative research projects. Education for information. 2004; 22(1):63–75.
50. Tong A, Sainsbury P, Craig J. Consolidated criteria for reporting qualitative research (COREQ): a 32-item checklist for interviews and focus groups. Int J Qual Health Care. 2007; 19(6):349–357. doi: 10.1093/intqhc/mzm042 PMID: 17872937
